# 738. Comparison of Characteristics of US International Travelers Seeking Pretravel Health Consultations at US Global TravEpiNet Sites Before and During the COVID-19 Pandemic

**DOI:** 10.1093/ofid/ofab466.935

**Published:** 2021-12-04

**Authors:** Mylinh H Le, Sowmya R Rao, Alison T Walker, Edward T Ryan, Regina C LaRocque, Emily P Hyle

**Affiliations:** 1 Medical Practice Evaluation Center, Boston, MA; 2 Massachusetts General Hospital, Boston, MA; 3 Centers for Disease Control and Prevention, Atlanta, GA

## Abstract

**Background:**

In January–March 2020, the Centers for Disease Control and Prevention (CDC) issued multiple warnings regarding COVID-19 travel-associated risks. We sought to describe US travelers seeking pretravel consultation regarding international travel at US Global TravEpiNet (GTEN) sites before and after the initial COVID-19 travel warnings.

**Methods:**

We prospectively collected data at 22 GTEN sites pre-COVID-19 (January–December 2019) and 18 GTEN sites during the COVID-19 pandemic (April 2020–March 2021). We excluded travelers evaluated during January–March 2020, when CDC travel guidance was evolving rapidly. Travelers used standardized questionnaires to self-report data regarding demographics and travel-related characteristics. Providers confirmed these data and documented their recommendations during pretravel consultation, which could be performed virtually. We conducted descriptive analyses of differences in demographics, travel-related characteristics, vaccinations, and medications (SAS v9.4; Cary, NC).

**Results:**

Compared with 16,903 pre-COVID-19 consultations, only 1,564 consultations occurred during the COVID-19 pandemic, a 90% reduction (Table). During COVID-19, a greater proportion of travelers were children aged 1–5 years, visiting friends and relatives (VFR), with itineraries ≥ 30 days, and going to Africa; a smaller proportion of travelers were aged > 55 years, or traveling to Southeast Asia or the Western Pacific. During COVID-19, fewer vaccine-eligible travelers received vaccines at the pretravel consultation except for yellow fever, and a greater proportion were referred to another provider for vaccination (Figure).

Table. Demographics and travel-related characteristics of international travelers seeking pretravel consultation at Global TravEpiNet sites before and during the COVID-19 pandemic

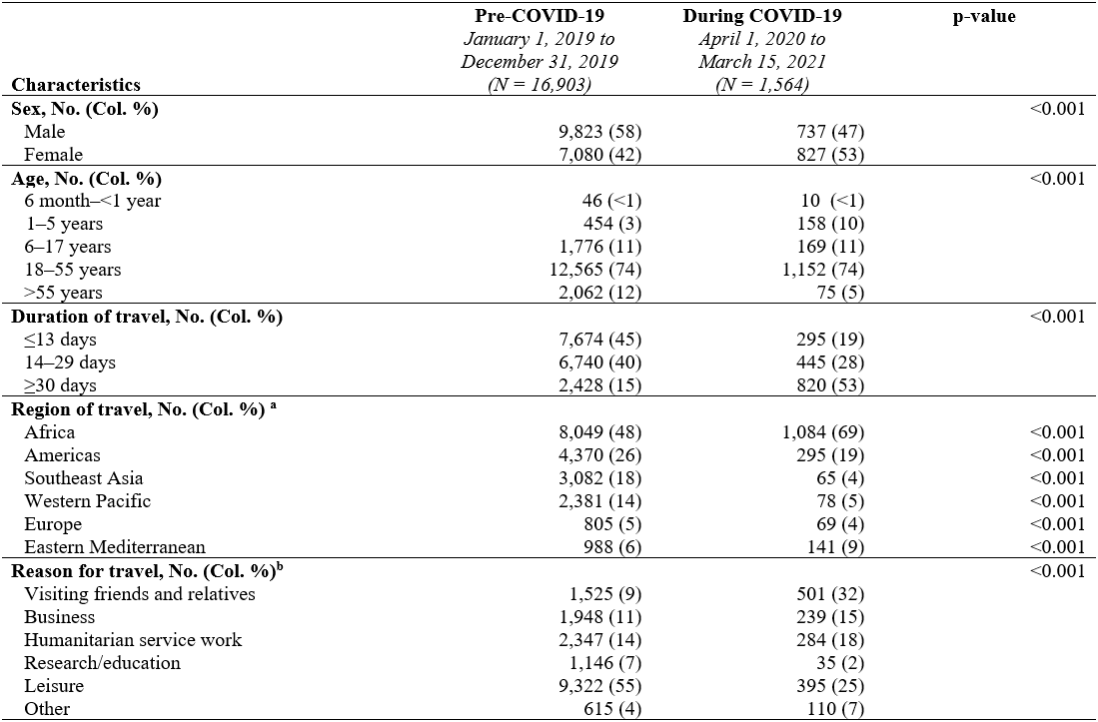

Table continued. Demographics and travel-related characteristics of international travelers seeking pretravel consultation at Global TravEpiNet sites before and during the COVID-19 pandemic

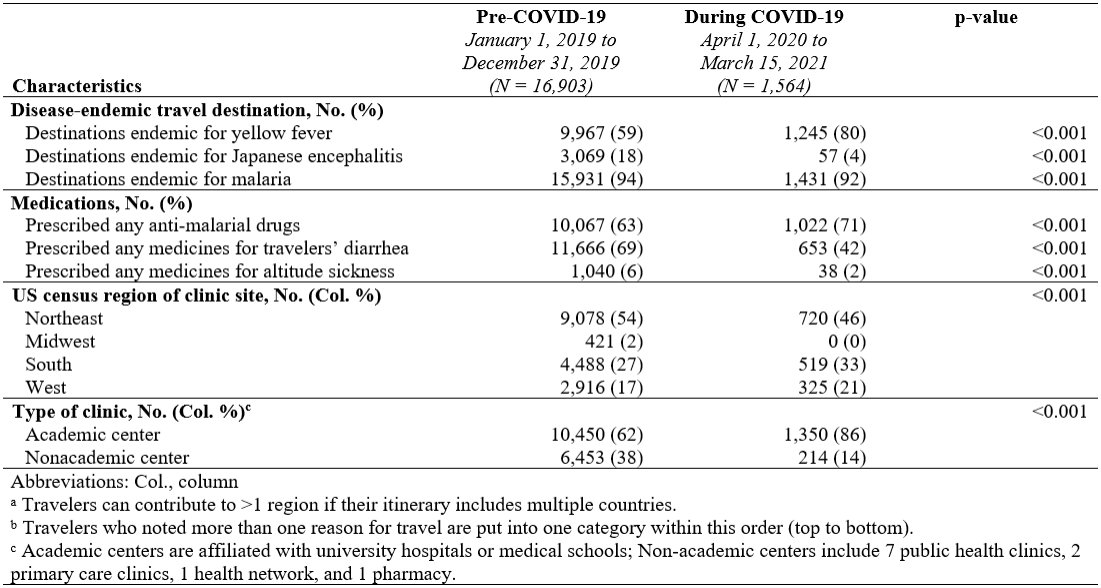

Figure. Vaccinations and reasons for nonvaccination among vaccine-eligible international travelers at pretravel consultations at Global TravEpiNet (GTEN) sites before and during the COVID-19 pandemic.

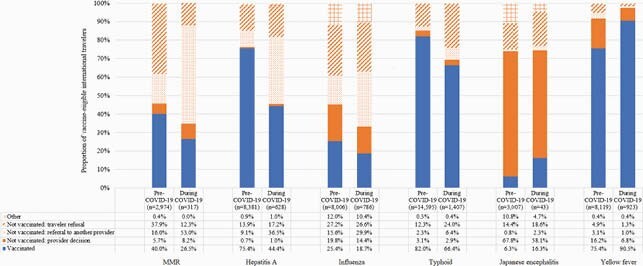

Among vaccine-eligible travelers, we summarized those who were vaccinated at the visit (blue) and not vaccinated (orange). We then categorized reasons for nonvaccination into: provider decision (solid), referral to another provider (dots), traveler refusal (striped), or other (hatched). COVID-19 vaccination was not available at GTEN sites during the analysis period; although COVID-19 vaccinations outside of GTEN sites might have affected vaccination recommendations, they were unlikely to have had a large effect given their limited availability in January-March 2021.

**Conclusion:**

Compared with pre-COVID-19, US travelers seeking pretravel consultations at GTEN sites during the pandemic might be at higher risk for travel-related infections given VFR status, traveling for ≥ 30 days, and going to Africa. Fewer vaccine-eligible travelers were vaccinated at pretravel consultations, which could reflect more virtual pretravel consultations. Counseling and vaccination for international travelers continue to be priorities during the COVID-19 pandemic.

**Disclosures:**

**All Authors**: No reported disclosures

